# Local Anesthesia in Piglets Undergoing Castration—A Comparative Study to Investigate the Analgesic Effects of Four Local Anesthetics Based on Defensive Behavior and Side Effects

**DOI:** 10.3390/ani10101752

**Published:** 2020-09-26

**Authors:** Nora Abendschön, Steffanie Senf, Pauline Deffner, Regina Miller, Andrea Grott, Julia Werner, Anna M. Saller, Judith Reiser, Christine Weiß, Yury Zablotski, Johannes Fischer, Shana Bergmann, Michael H. Erhard, Christine Baumgartner, Mathias Ritzmann, Susanne Zöls

**Affiliations:** 1 Clinic for Swine, Center for Clinical Veterinary Medicine, LMU Munich, 85764 Oberschleißheim, Bavaria, Germany; S.Senf@med.vetmed.uni-muenchen.de (S.S.); P.Deffner@med.vetmed.uni-muenchen.de (P.D.); C.Weiss@med.vetmed.uni-muenchen.de (C.W.); Y.Zablotski@med.vetmed.uni-muenchen.de (Y.Z.); Ritzmann@med.vetmed.uni-muenchen.de (M.R.); S.Zoels@med.vetmed.uni-muenchen.de (S.Z.); 2 Chair of Animal Welfare, Ethology, Animal Hygiene and Husbandry, LMU Munich, 80539 Munich, Bavaria, Germany; r.miller@tierhyg.vetmed.uni-muenchen.de (R.M.); a.schoerwerth@tierhyg.vetmed.uni-muenchen.de (A.G.); s.bergmann@tierhyg.vetmed.uni-muenchen.de (S.B.); m.erhard@tierhyg.vetmed.uni-muenchen.de (M.H.E.); 3 Center of Preclinical Research, Technical University of Munich, 81675 Munich, Bavaria, Germany; julia.werner@tum.de (J.W.); anna.saller@tum.de (A.M.S.); judith.reiser@tum.de (J.R.); fischer.johannes@tum.de (J.F.); christine.baumgartner@tum.de (C.B.)

**Keywords:** piglet, castration, local anesthesia, behavior, pain, procaine, lidocaine, bupivacaine, mepivacaine, animal welfare

## Abstract

**Simple Summary:**

More than 80 million male piglets are castrated every year within the first week of life mostly without pain relief in the EU. Castration is performed to prevent boar taint, to minimize aggressive and sexual behavior associated with intact males and to gain a constant quality of meat. It is an important animal welfare issue to eliminate pain caused by castration. Local anesthesia, meaning the sole injection of a local anesthetic into the testicles and in the surrounding tissue of conscious piglets, prior to castration is a currently discussed method in Germany. Thus, it was the aim of the present study to investigate the effect of four local anesthetics (procaine, lidocaine, bupivacaine and mepivacaine) on pain relief during surgical castration in conscious piglets. To assess pain, defensive behavior of piglets undergoing injection and castration was evaluated. In addition, attention was paid to possible side effects. Considering healing, bleeding and weight gain no negative impacts were observed, but impairments of locomotor activity occurred. The results indicate that lidocaine and mepivacaine are able to achieve significant pain relief during the castration procedure, whereas procaine and bupivacaine only during the severing of the spermatic cord.

**Abstract:**

The aim of the present study was to investigate the effect of four local anesthetics on pain relief during surgical castration under standardized conditions in conscious piglets. Therefore, 71 male piglets (three to seven days) were distributed into control groups (handling, castration without anesthesia or analgesia) and local anesthetic trial groups (procaine, lidocaine, bupivacaine, mepivacaine). Then, 20 min prior to castration, animals of the treatment groups, except piglets in the handling group, received an injection of a local anesthetic or sodium chloride of 0.5 mL intratesticularly and 0.5 mL subscrotally. During injection and castration, defensive behavior was evaluated. Locomotor activity, as well as postoperative bleeding, wound healing and average daily weight gain were assessed to detect side effects. The injection caused increased defensive movements, significantly in the bupivacaine group. Lidocaine and mepivacaine significantly reduced defensive movements during castration, and procaine and bupivacaine only during severing of the spermatic cord. Impairments of locomotor activity were found in piglets injected with lidocaine, bupivacaine or sodium chloride. Considering healing, bleeding and weight gain, no negative impacts were observed. In conclusion, lidocaine and mepivacaine were able to achieve significant pain relief during the castration procedure, whereas procaine and bupivacaine only during the severing of the spermatic cord. Moreover, the injection of bupivacaine seemed to be painful itself.

## 1. Introduction

In the EU, more than 80 million male piglets are castrated every year within the first week of life, mostly without pain relief [1]. Castration is performed to prevent boar taint, to minimize aggressive and sexual behavior associated with intact males and to gain a constant quality of meat [2,3,4]. The majority of the EU member states are still carrying out surgical castration with or without anesthesia. The exceptions are Ireland, the United Kingdom, the Netherlands, Portugal and Spain, where 20% or less of the male pigs are castrated [5]. According to EU Directive 2008/120/EC, better practices are demanded [6]. In Germany, the Animal Welfare Act prohibits the surgical castration of male piglets without anesthesia, even under the age of eight days, as of 1 January 2021 [7]. In addition to a complete waiver of surgical castration by implementing the practices of boar fattening or GnRH (gonadotropin releasing hormone) vaccination, an improved practice of male piglet castration requires sufficient anesthesia and analgesia to avoid acute and postoperative pain. Several possibilities to ensure these requirements are discussed: injectable anesthesia, inhalation anesthesia and local anesthesia. The possibility of castration with local anesthetics is currently not permitted in Germany, because the evidence of the efficacy of this method is still controversially discussed. Kluivers-Poodt et al. [8], Hansson et al. [9] and Leidig et al. [10] concluded that the use of local anesthetics reduces pain responses during castration, whereas Perez-Pedraza et al. [11] did not show any effect. Bonastre et al. [12] as well as Rauh et al. [13] indicated that local anesthetics lead to a reduction in pain-related responses, without eliminating the pain. These previous studies differ in terms of used local anesthetics, application methods and obtained pain associated parameters and are therefore difficult to compare. Procaine is the local anesthetic with the lowest anesthetic potency [14] and the only approved local anesthetic for pigs in Germany at the moment. Lidocaine is currently used for piglet castration in Norway, Sweden and Italy [5]. The effects of procaine and lidocaine in piglets undergoing castration have been investigated in various studies [8,9,10,11,12,13,15] but showed contradictory results. Furthermore, Bonastre et al. [12] investigated the effect of the combination of lidocaine and bupivacaine. Bupivacaine is described with the highest anesthetic potency [14]. The local anesthetic mepivacaine is commonly used in horses. To the authors’ knowledge, there has been no investigation on mepivacaine in conscious piglets undergoing castration so far.

To assess pain in piglets undergoing castration, different methods are available. As physiological parametersi.e., blood parameters, such as cortisol [12,16,17,18,19,20] adrenalin and adrenocorticotropic hormones [16,18,19] and changes in heart rate and blood pressure [21] can be assessed. Furthermore, behavioral variables, such as defensive movements [10,21,22] and vocalization [8,20,23,24,25,26,27] can be used for pain assessment. Additionally, Bilsborrow et al. [28] described an objective test to assess pain in piglets where the navigation time through a handling chute is determined.

In a first already published study part, the four local anesthetics, procaine, lidocaine, bupivacaine and mepivacaine, were evaluated based on pain-related physiological parameters and limb movements under a minimal anesthesia model using low doses of isoflurane [21]. Under light anesthesia, all four local anesthetics were highly effective at reducing signs of nociception during castration.

The aim of the present study was to investigate the effect of four local anesthetics (procaine, lidocaine, bupivacaine, mepivacaine) on pain relief during surgical castration under standardized conditions in conscious piglets as a second part of a comprehensive study. To evaluate pain, defensive movements and vocalization were assessed. To detect side effects locomotor activity, postoperative bleeding, wound healing, weight gain and mortality were evaluated.

## 2. Materials and Methods

Data of four groups of piglets castrated under local anesthesia were collected and compared with piglets castrated without local anesthesia and with only handled piglets. The study was performed in accordance with the EU Directive 2010/63/EU on the protection of animals used for scientific purposes and the German Animal Welfare Act (2019). The research protocol was approved by the Ethical Committee for Animal Experiments of the Government of Upper Bavaria, Munich, Germany (reference number ROB-55.2-2532.Vet_02-19-11).

### 2.1. Animals and Housing

The present randomized, double-blinded study was conducted in an experimental farrow-to-finish farm in Bavaria, Germany, with 80 sows farrowing in a three-week batch. The piglets (Piétrain × Large White/Landrace) were housed in farrowing units on partially slatted floor, except for the nest area, which was made up of concrete floor and provided with shavings and an infrared heat lamp. All piglets in the study received 1 mL of an intramuscular iron supplementation (Ursoferran 200 mg/mL solution for injection for pigs, Serumwerk Bernburg AG, Bernburg, Germany) the day before castration. Besides, no other painful procedures, such as tail docking or ear tagging, were carried out before the start of the study.

Data were collected from 71 male piglets between three to seven days of life (5.2 ± 1.09 days, 2.2 ± 0.48 kg (mean ± standard deviation)) of 16 litters. Inclusion criteria were a good general condition, a healthy sow and a minimum weight of 1.4 kg on the day of castration. Piglets with any deviation from the normal anatomical condition (e.g., hernia scrotalis or inguinalis) were excluded.

### 2.2. Experimental Design

**Figure 1:**
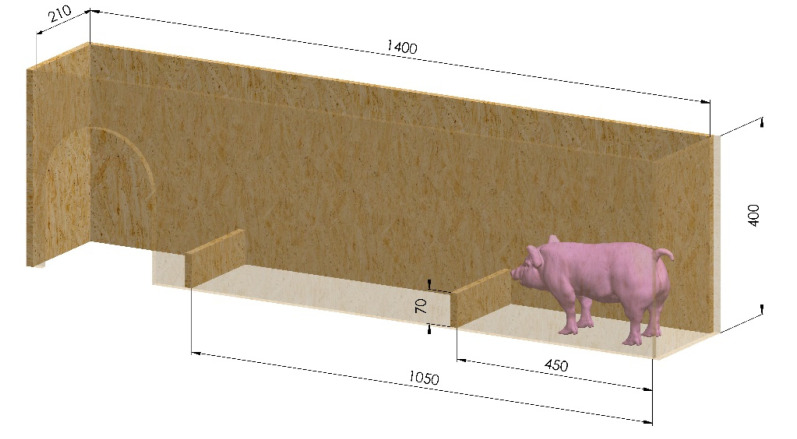
Illustration of the handling chute according to Bilsborrow et al. [28]; dimensions = internal dimensions in mm.

On the day before castration, each piglet was weighed (MS weighing plateau max. 100 kg, Schippers GmbH, Kerken, Germany), the general conditions of the piglets were checked and a computer-generated simple randomization was used to distribute the animals to one of the six experimental groups (Table 1). Additionally, the handling chute, according to Bilsborrow et al. [28] was trained four times with each piglet (two times without and two times with hurdles) (Figure 1).

On the morning of the castration day, piglets passed through the handling chute three consecutive times to determine the baseline. During injection and castration, all piglets, including the handling group, were fixed in a castration cradle (Schippers GmbH, Kerken, Germany). The piglets were given at least five minutes to rest before the injection.

All piglets except those of the handling group (H, only fixation) were injected with a total volume of 2 mL with the respective local anesthetic (procaine hydrochloride (P), lidocaine hydrochloride (L), bupivacaine hydrochloride (B) and mepivacaine hydrochloride (M)) or 0.9% sodium chloride (NaCl) according to the study group. Then, 0.5 mL were injected intratesticularly and 0.5 mL subscrotally for each testicle. For the injection an automatic self–filling system 1 mL syringe (HSW ECO–MATIC^®^, Henke-Sass, Wolf GmbH, Tuttlingen, Germany) with a 25 G sized cannula (0.5 × 16 mm, B. Braun TravaCare GmbH, Hallbergmoos, Germany) was used. The intratesticular injection was performed by fixing the testicle between the thumb and index finger caudally. Directly following the intratesticular injection the subscrotal single point injection was performed by releasing the fixed testicle, retracting the cannula from it but leaving the cannula under the skin and making a skin fold of the scrotum. The intratesticular and subscrotal injection was repeated for the second testicle. Thereafter, the piglets were returned into the farrowing pen for a period of 20 min.

Afterwards, all piglets were removed again from the farrowing pen and, with the exception of group H (only fixation and simulated interventions), castrated. Therefore, the scrotal area was cleaned with an antiseptic solution (octenisept^®^ aqueous wound and mucous membrane antiseptic, colorless, Schuelke & Mayr GmbH, Norderstedt, Germany). Using a sterile scalpel (scalpel blades carbon steel, sterile 21, Heinz Herenz Medizinalbedarf GbmH, Hamburg, Germany; scalpel handle no. 4, AESCULAP AG & CO. KG, Tuttlingen, Germany) for every piglet, scrotal tissue and tunica vaginalis were opened by a skin incision parallel to the raphe scroti and the first testicle was removed by severing of the spermatic cord with the scalpel. Skin incision and severing of the spermatic cord were repeated for the second testicle. The injection and castration were performed by three trained veterinarians. After surgery, no disinfectant was applied on the scrotal area to avoid behavioral changes by irritating substances. Group NaCl served as positive control for pain-related behavior induced by injection and castration without pain relief. As negative control, piglets from group H underwent simulated injection and castration to measure stress related behavior. In order to avoid an analgesic effect on the pain-related behavior during and after injection and castration, meloxicam (0.4 mg/kg) (Metacam^®^ 5 mg/mL, solution for injection for cattle and pigs, Boehringer Ingelheim Pharma GmbH & Co. KG, Ingelheim am Rhein, Germany) was not administered until one day after castration.

### 2.3. Data Sampling

For data sampling, evaluation of recordings and also data analysis for all persons involved were blinded at any time with the exception for group H (handling).

#### 2.3.1. Defensive Behavior

To evaluate defensive movements, piglets were filmed during injection and castration procedure (Samsung Galaxy S6-Samsung Electronics GmbH, Schwalbach, Germany). Analyzing of the defensive movements according to a score modified by Leidig et al. [10] (Table S1) was always performed by the same blinded person. “Injection”, “skin incision” and “severing of the spermatic cord” were evaluated separately according to intensity (0–4) and duration (0–3). The intensity was scaled into 0 = no movements, 1 = moving of one limb, 2 = moving of two limbs, 3 = moving of three limbs, 4 = moving of all four limbs. The duration was scaled into 0 = no movements, 1 = one single movement, 2 = repeated movements (2–4), 3 = continuous movements (>4). Defensive movements were evaluated for the right and left testicle separately. The single scores resulted in a maximum of seven for each testicle and step. The scores of both testicles and each step were summed up for evaluation. This results in a combined total score for “injection”, “skin incision” and “severing of the spermatic cord” from 0 to 14. On the basis of the audio of these recordings, a blinded person evaluated vocalization with “Yes” or “No”. Vocalization was assessed with “Yes”, if there was an acute onset of increased vocalization during the injection and/or castration procedure. Moreover, two additional blinded persons evaluated the recordings of defensive movements independently to ensure the reliability of the observer.

#### 2.3.2. Handling Chute Behavior

To evaluate the navigation time according to Bilsborrow et al. [28], piglets passed through the handling chute after injection and castration, two hours after castration and one day after castration. The handling chute had a length of 1400 mm, width of 210 mm, height of 400 mm, two 70 mm hurdles (distance between the hurdles: 600 mm) and an open top to record the duration and quality of the run (Panasonic LUMIX DMC-FZ8, Matsushita Electric Industrial Co., Ltd., Osaka, Japan) (Figure 1). Additionally, a structured thermal mat was used as a base for the handling chute to avoid slipping. The navigation time was determined, based on the recordings, starting when the piglets began running and ending when all four limbs were over the last hurdle. Particular attention was paid to impairments of the locomotor activity (unsteady gait, stumble upon the hurdles, sitting/lying in the handling chute).

#### 2.3.3. Postoperative Assessment

Piglets were weighed and wound healing was scored according to Zankl [29] (Table S2) on days 1, 7, 14 and 21 after castration to assess the postoperative period up to day 21. Castration wounds were ranked on the basis of wound healing (0 = without specific findings (dry, fully closed wound), 1 = minor findings (swelling, redness), 2 = open wound), wound secretion (0 = no secretion, 1 = serous/bloody secretion, 2 = purulent secretion) and texture and size of the spermatic cord (0 = hardly palpable, 1 = up to 1 cm, soft to rough and elastic, 2 = stronger than 1cm, soft to rough and elastic, 3 = stronger than 1 cm, rough or fluctuating). The score was summed up for evaluation with a maximum of seven. Postoperative bleeding was quantified two hours after castration using a score adapted by Enz et al. [30] (Table S3). The score ranged from 0 (no bleeding) to a maximum of 3 (severe bleeding, perineal area and hind limbs bloodstained). In addition, mortality was recorded until day 21 post castration.

### 2.4. Statistical Analysis

For the statistical analysis, all experimental groups were tested for age and weight on day of castration, defensive movements, vocalization, navigation time, wound healing, postoperative bleeding, and average daily weight gain. The distribution of all continuous parameters was tested using Shapiro–Wilk normality test. For non-normally distributed parameters defensive movements, wound healing and postoperative bleeding a Kruskal–Wallis test was performed. Dunn test for multiple comparisons between the groups P, L, B, M, H and NaCl with Benjamin–Hochberg corrections followed the Kruskal–Wallis test. For normally distributed parameters (average daily weight gain) ANOVA was used with Student’s t-test for pairwise comparisons. Nonparametric bootstrap was used for obtaining confidence limits for the population mean without assuming normality. For mostly normal distributed parameters age and weight on day of castration a Mann–Whitney-U test was performed. Vocalization was analyzed using pairwise Fisher’s tests with Benjamin–Hochberg corrections. Navigation time was analyzed using mixed-effects linear model due to the presence of repeated measures, with time, group and interaction between them as fixed effects and a random effect of individual piglet. Due to the non-normality and heteroscedasticity of the residuals, the response variable was log-transformed.

Statistical significance was considered at *p* < 0.05. These statistical analyses were performed using R statistical software version 3.6.1, mainly with “ggstatsplot” [31], “lme4” [32] and “emmeans” [33] packages and IBM SPSS Statistics for Windows, Version 26.0 (IBM Corp., Armonk, NY, USA). For the score used to evaluate the defensive movements, the Intraclass Correlation Coefficients (ICC) and their 95% confident intervals were calculated using IBM SPSS Statistics for Windows, Version 26.0, based on mean rating, absolute agreement and 2-way mixed-effects model. The ICC values ranged between 0 and 1, with values closer to 1 representing stronger reliability.

## 3. Results

Day of castration varied between third and seventh day of life (dl) of the piglets and the mean age of the groups differed between 4.75 ± 0.97 dl (Mean ± standard deviation (SD)) in group NaCl and 5.58 ± 1.40 dl in group M at castration day (*p* ≥ 0.05). Mean body weight on day of castration differed significantly between piglets of group M (2.53 ± 0.59 kg) and groups L (2.08 ± 0.44 kg) (*p* < 0.05) and numerically between group M and B (2.09 ± 0.42 kg) (*p* < 0.1) (Table 2).

### 3.1. Defensive Behavior

**Figure 2:**
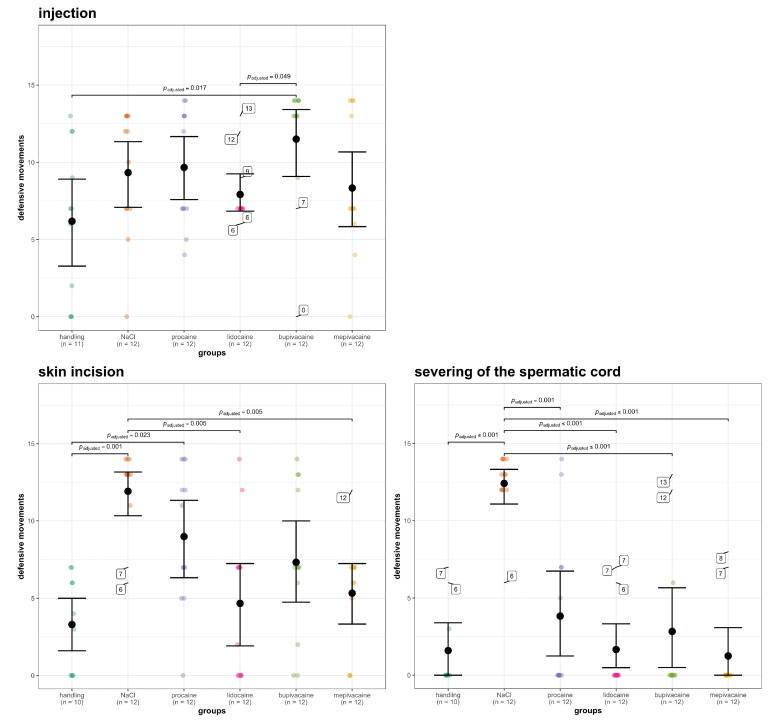
Mean values, confidence intervals and *p*-values of the score used to evaluate the defensive movements in different groups during injection, skin incision and severing of the spermatic cord; outliers were calculated using Tukey´s method and were tagged on the picture with a value instead of the data point to avoid clutter.

The Intraclass Correlation Coefficients (ICCs) for the evaluation of the defensive movements according to the modified score of Leidig et al. [10] were 0.97 (confidence interval (CI): 0.95–0.98) for injection, 0.96 (CI: 0.94–0.98) for skin incision and 0.98 (CI: 0.97–0.99) for severing of the spermatic cord. As shown in Figure 2, during injection, only group B showed significantly more defensive movements compared to groups H and L. Among the other groups, no significant difference could be detected during the injection. During skin incision groups L, M and H showed significantly fewer defensive movements compared to group NaCl. In groups P and B, no significant difference was found compared to group NaCl. Groups NaCl and P showed significantly more defensive movements compared to group H during skin incision. No significant difference was found between group B and the other groups during skin incision. The highest mean score of defensive movements was found in group NaCl during the severing of the spermatic cord. Regarding the severing of the spermatic cord, all local anesthetic groups (P, L, B, and M) and group H showed significantly fewer defensive movements compared to group NaCl. Apart from this, no differences among the groups were found during the severing of the spermatic cord.

During injection, no significant difference was found among the groups regarding vocalization. Three of the 10 piglets in group H showed an acute onset of increased vocalization during sham skin incision and sham severing of the spermatic cord in contrast to 11 of 12 piglets in group NaCl (*p* < 0.05), as shown in Table 3. During the severing of the spermatic cord, one piglet in group M showed an acute onset of increased vocalization in contrast to 11 in group NaCl (*p* < 0.05).

### 3.2. Handling Chute Behavior

#### 3.2.1. Navigation Time

As shown in Table 4, the baseline of navigation time of the groups differed between 5.5 ± 2.3 s (S) (mean ± SD) (NaCl) and 7.6 ± 4.1 s (L) (*p* ≥ 0.05). After injection, group M passed through the handling chute within 3.8 ± 1.8 s (fastest) and group L within 9.4 ± 7.6 s (slowest) (*p* < 0.05). After castration, the fastest navigation time was measured for group M with 3.5 ± 1.25 s and the slowest for group B with 8.9 ± 11.47 s (*p* < 0.05). Two hours after castration, group M navigated the handling chute within 2.4 ± 0.51 s, significantly faster than groups L (5.0 ± 5.41 s), B (4.5 ± 2.3 s) and P (4.1 ± 1.4 s) (*p* < 0.05). Regarding the navigation time, no significant difference between control groups NaCl and H could be detected at any time (*p* ≥ 0.05).

#### 3.2.2. Locomotor Activity

As shown in Table 5, after injection, castration and two hours after castration, an impairment of the locomotor activity occurred in groups NaCl, L (two piglets in each case) and B (three piglets). Two piglets died one day after castration.

### 3.3. Postoperative Assessment

#### 3.3.1. Wound Healing

One day after castration, one piglet in group B had a heightened wound healing score of three. On days 7, 14 and 21 after castration, the wound healing score of all piglets was below 3, without differing significantly between groups at any time.

#### 3.3.2. Postoperative Bleeding

Piglets of group NaCl showed the slightest postoperative bleeding score (1.25 ± 1.42 (Mean ± SD)). Postoperative bleeding scores of the local anesthetic groups were 2.08 ± 0.97 (P), 1.75 ± 1.42 (L), 1.67 ± 1.38 (B) and 2.08 ± 1.38 (M). No significant differences were found between the local anesthetic groups or between any of the local anesthetic groups and group NaCl.

#### 3.3.3. Mortality Rate and Average Daily Weight Gain

In total, three (4.2%) of 71 piglets died during the trial period from castration day until weaning. Two of them were from the same litter and died the same day after castration (groups NaCl and L). The piglet injected with lidocaine was examined histopathologically. The autopsy showed a severe hemorrhage into the abdomen. The histological examination of the lungs showed a hemorrhagic and necrotizing pneumonia. Another piglet (group NaCl) died between days two and seven after castration due to crushing by the sow.

No effect of treatment was found on average daily weight gain (ADW). The ADW of the piglets ranged between 0.131 and 0.353 kg, except for one outlier (0.075 kg) in group NaCl. Between groups, ADW differed between 0.195 ± 0.049 kg (Mean ± SD) in group L and 0.247 ± 0.036 kg in group M.

## 4. Discussion

Several studies have already been conducted on local anesthesia as an alternative to piglet castration without anesthesia but they differed in study designs, local anesthetics used and yielded heterogeneous results [8,9,10,11,12,13]. This second part of a comprehensive study examined the four local anesthetics, procaine, lidocaine, bupivacaine and mepivacaine, in conscious piglets during castration in the first week of life. Part one of the comprehensive study showed that the intratesticular injection with a subcutaneous depot of a local anesthetic prior to castration reduces nociception-related cardiovascular responses and limb movements in piglets under anesthesia [21].

Similar to Leidig et al. [10] and Rauh et al. [13], we used defensive movements as a suitable parameter to evaluate pain responses in conscious piglets. In the present study, the ICC values for the score used to evaluate defensive movements were more than 0.9 for “injection”, “skin incision” and “severing of the spermatic cord”, which indicates an excellent Interrater Reliability [34].

The highest mean score of defensive movements occurred in group NaCl (castration without anesthesia) during severing of the spermatic cord. This is in line with the findings of Rauh et al. [13] and thus it confirms the suggestion based on the vocalization of Taylor and Weary [35] that pulling and severing of the spermatic cords are the most painful components of castration. Almost the same score was observed in group NaCl during skin incision, which is consistent with several studies that the whole castration procedure without anesthesia causes severe pain [8,10,35,36]. Prior studies showed, based on defensive movements, that the application of lidocaine intratesticularly and subscrotally reduces pain responses during the castration procedure [9,13]. In the present study, the injection of procaine, lidocaine, bupivacaine or mepivacaine led to a significant reduction in defensive movements compared to castration without anesthesia during the severing of the spermatic cord, whereas during skin incision a significant reduction was observed only after the injection of lidocaine or mepivacaine. An explanation for the different effects of local anesthesia on skin incision and severing of the spermatic cord could be that with the subscrotal and intratesticular applicated depots used in the present study, the local anesthetics did not readily diffuse into the skin. Ranheim et al. [37] discovered, through autoradiograms, that radiolabeled lidocaine injected into the testis and subcutaneously into the scrotum does not readily diffuse through the tunica vaginalis. However, the assumption that the local anesthetics did not readily diffuse into the skin does not explain the different extent of the reduction in defensive movements between local anesthetics during skin incision. The injection of lidocaine and mepivacaine led to a significant reduction in defensive movements during skin incision, while procaine and bupivacaine application did not. Another possible explanation lies in the physiochemical properties of the used local anesthetics. Local anesthetics are weak bases and thus exist in equilibrium between the neutral, non-ionized, lipid-soluble and the ionized, water-soluble form. The position of equilibrium can be defined using pKa. The pKa of a molecule represents the pH at which 50% exists in the non-ionized (lipid-soluble) and 50% in the ionized form (water-soluble) [38]. The main access of local anesthetics to the cell is by penetration of the lipophilic neutral form through the lipid membrane [14,39]. Therefore, a local anesthetic with a low pKa (mepivacaine: 7.72; lidocaine: 7.77; bupivacaine: 8.1; procaine: 8.89) will have a greater proportion of the non-ionized lipid-soluble form at physiological pH, resulting in better diffusion into the surrounding tissue and a more rapid onset of action. Moreover, the relative anesthetic potency is of importance as well. The main determinant of local anesthetic potency is the lipid solubility [40,41]. However, we could not prove that the local anesthetic with the highest lipid solubility and the highest relative anesthetic potency, in this case bupivacaine, produced the greatest reduction in pain related behavior. According to these results, we assume that the spermatic cord was anesthetized more efficiently than the scrotal skin. For future research, optimizing the application method is needed to improve the anesthesia of the scrotal skin.

To evaluate pain caused by the injection, irrespective of the acidity of local anesthetic solutions, sodium chloride (NaCl) was injected into the testicles of the piglets castrated without anesthesia. In the present study, piglets that received an intratesticular and subscrotal injection tended to have higher scores of defensive movements and vocalization than piglets that were handled only (H). The lack of significance between the control groups H and NaCl could result from the stress and fear provoked by the fixation of the piglet in a supine position and the fixation of the testicles, which may have overlapped the defensive behavior caused by the injection. Moreover, piglets injected with bupivacaine showed significantly more defensive movements than piglets injected with lidocaine or piglets that were only fixated. However, this result is contradictory to the assumption that the pain induced by injection of local anesthetics is believed in part to be related to the acidity, since Bupivacain 0.5% with epinephrine 0.0005% JENAPHARM^®^ (mibe GmbH Arzneimittel, Brehna, Germany) and Xylocitin^®^ 2% (mibe GmbH Arzneimittel, Brehna, Germany) with epinephrine 0.001% have the same pH. The results of the present study indicate that the injection of bupivacaine seemed to be painful itself. Additionally, the sole fixation of the testicles might induce defensive movements, as seen in the handling group, but to a lesser extent compared to injecting any substances.

Weary et al. [23] validated vocal measures as a reliable indicator of acute pain due to castration. Several studies confirmed that the vocalization of piglets castrated without anesthesia differs from piglets that were sham castrated [24] or castrated with anesthesia [8,25,26]. White et al. [25] assessed vocalization during the castration procedure of piglets with frequency of highest energy (HEF). Weary et al. [23], Taylor et al. [24] and Puppe et al. [27] used high frequency calls. In contrast, Marx et al. [26] classified calls during castration into three different call types (grunt, squeal, scream) and demonstrated that piglets castrated without anesthesia produced almost twice as many screams as piglets castrated with local anesthesia. It is necessary to mention that, in their studies, for the recordings of the vocalization separate rooms were used to prevent interfering noises [23,26,27]. Furthermore, collected data were elaborately analyzed with special programs [23,24,25,26,27]. In the present study, vocalizations were recorded in the nursery and not in a separate room, so the occurrence of interfering noises could not be prevented. As a practicable method to assess vocalization, despite the interfering noises, an acute onset of increased vocalization, comparable with the call type “scream” was evaluated in the present study, in orientation to the classification of Marx et al. [26]. Since no computer program was used for the evaluation of the vocalization in the present study, it is difficult to compare the results with prior studies. Nonetheless the results obtained are similar to former studies [8,10,23,24,25,26,35]. Piglets castrated without anesthesia (NaCl) reacted significantly more often with an acute onset of increased vocalization compared to piglets that were handled (H). In contrast to Marx et al. [26] and Kluivers-Poodt et al. [8] no significant difference occurred between the control groups and piglets castrated with local anesthesia during the castration procedure, with the exception of piglets injected with mepivacaine during the severing of the spermatic cord. This could be due to the used measurement of vocalization in the present study and the resulting lower sensitivity.

The navigation time through the handling chute was determined with the assumption that the presence of pain would result in longer navigation times [13,28]. According to Bilsborrow et al. [28], piglets castrated without anesthesia had a significantly longer navigation time through a handling chute 0 and 15 min after castration than sham castrated piglets [28]. Davis et al. [42] and Rauh et al. [13] validated these results. However, no significant difference regarding navigation time was found between control groups castrated without anesthesia and sham castrated in the present study. In contrast, piglets injected with mepivacaine had a significantly shorter navigation time after injection, castration and two hours after castration, possibly because of significant weight differences. Piglets injected with mepivacaine were significantly heavier. The significant differences regarding the weight on day of castration could be caused by the small number of piglets. Since no significant difference was found between both control groups in the present study, the handling chute was not suitable to assess pain objectively following castration in the present study. However, the recordings were not only used to measure navigation time, but also to get evidence of impairments of locomotor activity. The impairments occurred, as shown in Table 5, as an unsteady gait, kyphotic spine, tripping, sitting or lying in the handling chute or refusing the hurdles. Two piglets injected with bupivacaine showed such impairments after injection. This is in line with the results of defensive movements, where piglets injected with bupivacaine showed the highest score during injection. However, we could not explain these results. Furthermore, after castration, one piglet injected with sodium chloride, one piglet injected with lidocaine and two piglets injected with bupivacaine showed impairments. In addition, one piglet injected with bupivacaine showed an impairment two hours after castration. The impairments of individual piglets of groups NaCl, L and B after castration could be explained by pain itself. Moreover, local anesthetics could cause side effects, such as impairments of locomotor activity. Since every piglet was injected with a standardized dosage of local anesthetic regardless of its weight, an overdose in lighter piglets might be conceivable. Furthermore, it is possible that heavier piglets had a loss of effectiveness, although we could not observe an effect. However, the piglets concerned were not among the lightest. Further studies are necessary to investigate how different dosages, based on individual weights, influence the effectiveness of local anesthesia and the occurrence of side effects. Moreover, an inadvertent intravenous injection of the local anesthetics could result in central nervous system and cardiovascular toxicity. Lipid-soluble local anesthetics—i.e., bupivacaine followed by lidocaine or mepivacaine—are more potent and therefore cause more systemic toxicity than less lipid-soluble agents, such as procaine, which is described with a minimal potential for systemic toxicity [14]. Two piglets of the same litter (group NaCl and group L) stood out when they were supposed to complete the handling chute two hours after castration. Their general condition was severely disturbed two hours after castration and they were unable to walk or stand. Both piglets died the same day. Prior studies on local anesthesia, which applied lidocaine [8,9,12,15] or procaine [15], did not show any effect on mortality due to local anesthesia. Bonastre et al. [12] also did not show any effect on mortality caused by the local anesthetics, lidocaine and bupivacaine. Prior to the present study, no data were available on the mortality of piglets that were injected with the local anesthetic mepivacaine prior to castration. In the present study, the deceased lidocaine injected piglet was examined histopathologically. The autopsy showed a severe hemorrhage into the abdomen, probably associated with an increased bleeding propensity. Furthermore, we cannot exclude that the severe hemorrhage into the abdomen could have been caused by the surgical castration itself. The histology of the lungs showed a hemorrhagic and necrotizing pneumonia, which is most likely compatible with an infarction. Thus, in addition to the bleeding-induced loss of procoagulatory substances, an iatrogenic introduction of local anesthetic into the bloodstream with thrombus formation and a pronounced cardiotoxicity should also be considered. Since one piglet of group NaCl died as well, and both piglets were from the same litter, it is more likely that a coagulopathy may have caused their death. Unrestricted locomotor activity, especially in suckling piglets, is essential in order to avoid losses for example due to crushing by the sow. Moreover, it can be a sign of side effect of the injected bupivacaine or lidocaine. Further research is needed to investigate if it is possible to reduce the dosage without reducing the effect of the anesthesia.

As described by Hofmann et al. [15] and Zankl et al. [43], local anesthetics have no impact on wound healing after castration. In the present study, only one piglet injected with bupivacaine showed a heightened score one day after castration. This piglet defecated during castration, which possibly led to a contamination of the wound and consequently to an increased wound score, demonstrating that the castration should be performed as sterile as possible.

In the present study piglets were castrated only using a scalpel and no severe postoperative bleeding occurred in the scrotal area. The use of local anesthetics did not influence bleeding score two hours after castration, though local anesthetics contain different amounts of epinephrine or none (Mepidor 20 mg/mL). It was noticed that piglets castrated without any pain relief ranked the lowest considering postoperative bleeding. An explanation could be that, due to the pain caused by castration without pain relief, the adrenaline, which was released from adrenal glands, led to a greater vasoconstriction than the epinephrine, which is contained in the drugs. Moreover, neither castration without anesthesia nor castration with local anesthesia had a negative impact on daily growth performance until 21 days after castration. This is in line with the findings of Kluivers-Poodt et al. [8] and Bonastre et al. [12], who described that neither anesthetics nor meloxicam have an effect on mortality or growth performance in piglets. In contrast, Telles et al. [44] demonstrated that the use of local anesthetic prior to castration appears to have positive effects on long-term weight gain of pigs. Whether local anesthesia has an influence on long-term weight gain could not be evaluated in the present study, because the weights of the animals were only recorded until the 21st day after castration.

Limitations of the present study are the different piglet ages and weights on day of castration, use of commercially available local anesthetic products with different concentrations of epinephrine or no epinephrine at all, and the fact that the dosage was based on volume and not on weight. Furthermore, the small sample size should be mentioned as a limitation of the study.

## 5. Conclusions

It can be concluded that the local anesthetics lidocaine and mepivacaine were able to achieve significant pain relief during skin incision and during severing of the spermatic cord under standardized conditions in conscious piglets. The subscrotally and intratesticularly applicated local anesthetics procaine and bupivacaine could only relieve pain significantly during severing of the spermatic cord but not during skin incision. Moreover, due to the significantly higher occurrence of defensive movements the intratesticular and subscrotal injection of bupivacaine seemed to cause pain itself. Nonetheless, impairments of locomotor activity after the application of lidocaine, bupivacaine and sodium chloride occurred. Injection of the local anesthetics did not cause negative impacts on general condition, wound healing, weight gain or bleeding of the piglets after castration.

## Figures and Tables

**Table 1:** Classification of study groups.

Group	Preparation	Active Agent		Intervention		Animals (n)
		LA (mg/Animal)	Epin. (mg/mL)	Injection	Castration	
P	Pronestesic 40 mg/mL ^1^	procaine (80)	0.02	+	+	12
L	Xylocitin^®^ 2% ^2^	lidocaine (40)	0.01	+	+	12
B	Bupivacain 0.5% ^3^	bupivacaine (10)	0.005	+	+	12
M	Mepidor 20 mg/mL ^4^	mepivacaine (40)	-	+	+	12
NaCl	NaCl 0.9% ^5^	-	-	+	+	12
H	-	-	-	sham injection	sham castration	11

P = procaine; L = lidocaine; B = bupivacaine; M = mepivacaine; NaCl = sodium chloride; H = handling; ^1^ Pronestesic 40 mg/mL solution for injection for horses, cattle, pigs and sheep FATRO S.p.A, Ozzano Emilia (Bologna), Italy; ^2^ Xylocitin^®^ 2% with epinephrine, mibe GmbH Arzneimittel, Brehna, Germany; ^3^ Bupivacain 0.5% with epinephrine, JENAPHARM^®^, mibe GmbH Arzneimittel, Brehna, Germany; ^4^ Mepidor 20 mg/mL solution for injection for horses, Richter Pharma AG, Wels, Austria; ^5^ NaCl 0.9%, 0.9% Sodium Chloride Intravenous Infusion B. Braun Melsungen AG, Melsungen, Germany; LA: local anesthetic agent with hydrochloride; epin.: epinephrine.

**Table 2:** Mean values and standard deviation of age and body weight on day of castration.

Group	Age on Day of Castration		Weight on Day of Castration	
	Mean Value	SD	Mean Value	SD
procaine n = 12	5.00 dl	±0.95	2.23 kg	±0.44
lidocaine n = 12	5.58 dl	±0.90	2.08 kg ^a^	±0.44
bupivacaine n = 12	5.17 dl	±1.27	2.09 kg	±0.42
mepivacaine n = 12	5.58 dl	±1.34	2.53 kg ^b^	±0.59
NaCl n = 12	4.75 dl	±0.97	2.14 kg	±0.44
handling n = 11	5.09 dl	±0.94	2.32 kg	±0.45

Differing superscripts within a column indicate significant (*p* < 0.05) differences between groups; n = animals; dl = day of life; SD = standard deviation.

**Table 3:** Number of piglets with increased vocalization/number of piglets per group during injection, skin incision and severing spermatic cord.

Group	Number of Piglets with Increased Vocalization		
	Injection	Skin Incision	Severing Spermatic Cord
handling	6/10	3/10 ^a^	3/10 ^a^
NaCl	10/12	11/12 ^b^	11/12 ^b^
procaine	11/12	9/12	6/12
lidocaine	9/12	6/12	3/12
bupivacaine	10/12	9/12	3/12
mepivacaine	8/12	8/12	1/12 ^a^
*p*	n.s.	<0.05	<0.05

Differing superscripts within a column indicate significant (*p* < 0.05) differences between groups; n.s. = not significant.

**Table 4:** Mean values and standard deviation of navigation time in seconds of the piglets through the handling chute according to Bilsborrow et al. [28] in different groups before injection, after injection, after castration, two hours after castration and one day after castration.

Group	Injection		Castration		
	Before ^1^	After	After	2 h After	1 Day After
Procaine n = 12	6.02 ± 3.90	5.39 ± 3.12	6.78 ± 2.78 ^a^	4.12 ± 1.40 ^b^	3.38 ± 0.74
Lidocaine n = 12	7.64 ± 4.07 ^x^	9.50 ± 7.56 ^a^	6.40 ± 4.40	4.97 ± 5.41 ^x b^	4.45 ± 2.75 ^x^
Bupivacaine n = 12	6.46 ± 2.79	5.90 ± 3.76 ^x^	8.97 ± 11.47 ^x^	4.51 ± 2.29 ^b^	4.56 ± 2.33
Mepivacaine n = 12	5.86 ± 2.26	3.82 ± 1.79 ^b^	3.49 ± 1.25 ^b^	2.41 ± 0.51 ^a^	3.21 ± 1.18
NaCl n = 12	5.53 ± 2.26 ^x^	4.51 ± 1.39	4.13 ± 1.11 ^x^	3.70 ± 1.57 ^x^	2.85 ± 0.56 ^x^
Handling n = 11	6.09 ± 1.96	4.61 ± 1.36	5.39 ± 3.97	3.24 ± 0.99 ^xx^	3.88 ± 2.13
*p*	n.s.	<0.05	<0.05	<0.05	n.s.

Differing subscripts within a column indicate significant (*p* < 0.05) differences between groups; n = animals; ^x^ n = 11; ^xx^ = n = 10; ^1^ mean value from three consecutive runs; h = hours; n.s. = not significant.

**Table 5:** Impairments of locomotor activity at different times of individual piglets.

Group	Piglet	Weight in kg	Injection		Castration	
			After	After	2 h After	1 Day after
NaCl ^1^ n = 12	1	1.8		refusing the hurdles		
	2	2.9			not able to walk or stand	dead
L ^2^ n = 12	1	1.85			not able to walk or stand	dead
	2	1.8		unsteady gait		
B ^3^ n = 12	1	1.4	tripping, lying, kyph. spine	tripping		
	2	2.35		unst. gait, ref. the hurdles		
	3	2.5	unsteady gait		unsteady gait	

^1^ Injection of sodium chloride; ^2^ injection of lidocaine; ^3^ injection of bupivacaine; n = animals/group; kyph. = kyphotic; unst. = unsteady; ref. = refusing; h = hours.

## Supplementary Materials

The following are available online at https://www.mdpi.com/2076-2615/10/10/1752/s1, Table S1: Score used to evaluate the defensive movements according to Leidig et al. [10] during injection, skin incision and severing of the spermatic cord; Table S2: Score used to evaluate the castration wounds according to Zankl [29]; Table S3: Score used to evaluate the postoperative bleeding according to Enz et al. [30].

## References

[1] Fredriksen B., Font I.F.M., Lundstrom K., Migdal W., Prunier A., Tuyttens F.A., Bonneau M. (2009). Practice on castration of piglets in Europe. Animal.

[2] Rydhmer L., Zamaratskaia G., Andersson H., Algers B., Guillemet R., Lundström K. (2006). Aggressive and sexual behaviour of growing and finishing pigs reared in groups, without castration. Acta Agric. Scand. A Anim. Sci..

[3] Barton-Gade P.A. (1987). Meat and fat quality in boars, castrates and gilts. Livest. Prod. Sci..

[4] Bünger B., Schrader L., Schrade H., Zacharias B. (2015). Agonistic behaviour, skin lesions and activity pattern of entire male, female and castrated male finishing pigs. Appl. Anim. Behav. Sci..

[5] De Briyne N., Berg C., Blaha T., Temple D. (2016). Pig castration: Will the EU manage to ban pig castration by 2018?. Porcine Health Manag..

[6] Official Journal of the European Union COUNCIL DIRECTIVE 2008/120/EC of 18 December 2008 Laying down Minimum Standards for the Protection of Pigs. https://eur-lex.europa.eu/legal-content/EN/TXT/HTML/?uri=CELEX:32008L0120&from=EN.

[7] Federal Ministry of Justice and Consumer Protection Tierschutzgesetz. https://www.gesetze-im-internet.de/tierschg/BJNR012770972.html.

[8] Kluivers-Poodt M., Houx B.B., Robben S.R.M., Koop G., Lambooij E., Hellebrekers L.J. (2012). Effects of a local anaesthetic and NSAID in castration of piglets, on the acute pain responses, growth and mortality. Animal.

[9] Hansson M., Lundeheim N., Nyman G., Johansson G. (2011). Effect of local anaesthesia and/or analgesia on pain responses induced by piglet castration. Acta Vet. Scand..

[10] Leidig M.S., Hertrampf B., Failing K., Schumann A., Reiner G. (2009). Pain and discomfort in male piglets during surgical castration with and without local anaesthesia as determined by vocalisation and defence behaviour. Appl. Anim. Behav. Sci..

[11] Perez-Pedraza E., Mota-Rojas D., Ramirez-Necoechea R., Guerrero-Legarreta I., Martinez-Burnes J., Lezama-Garcia K., Mora-Medina P., Rosas M., Martinez V., Gonzalez-Lozano M. (2018). Effect of the number of incisions and use of local anesthesia on the physiological indicators of surgically-castrated piglets. Int. J. Vet. Sci. Med..

[12] Bonastre C., Mitjana O., Tejedor M.T., Calavia M., Yuste A.G., Ubeda J.L., Falceto M.V. (2016). Acute physiological responses to castration-related pain in piglets: The effect of two local anesthetics with or without meloxicam. Animal.

[13] Rauh A., Hofmann K., Harlizius J., Weiss C., Numberger J., Scholz T., Schulze-Horsel T., Otten W., Ritzmann M., Zöls S. (2019). Pain and distress response of suckling piglets to injection and castration under local anaesthesia with procaine and licocaine—Part 2: Defence behaviour, catecholamines, coordination of movements. Tierarztl. Prax. Ausg. G Grosstiere Nutztiere.

[14] Garcia E.R., Grimm K.A., Lamont L.A., Tranquilli W.J., Greene S.A., Robertson S.A. (2015). Local anesthetics. Veterinary Anesthesia and Analgesia: The Fifth Edition of Lumb and Jones.

[15] Hofmann K., Rauh A., Harlizius J., Weiss C., Scholz T., Schulze-Horsel T., Escribano D., Ritzmann M., Zols S. (2019). Pain and distress responses of suckling piglets to injection and castration under local anaesthesia with procaine and lidocaine—Part 1: Cortisol, chromogranin A, wound healing, weights, losses. Tierarztl. Prax. Ausg. G Grosstiere Nutztiere.

[16] Prunier A., Mounier A., Hay M. (2005). Effects of castration, tooth resection, or tail docking on plasma metabolites and stress hormones in young pigs. J. Anim. Sci..

[17] Zoels S., Ritzmann M., Heinritzi K. (2006). Effect of analgesics on the castration of male piglets. Berl. Munch. Tierarztl. Wochenschr..

[18] Schwab S., Follrich B., Kurtev V., Keita A. (2012). Ketoprofen-practical use and efficacy for post-surgical analgesia in piglet castration. Tierarztl. Umsch..

[19] Gottardo F., Scollo A., Contiero B., Ravagnani A., Tavella G., Bernardini D., De Benedictis G., Edwards S. (2016). Pain alleviation during castration of piglets: A comparative study of different farm options. J. Anim. Sci..

[20] Marchant-Forde J., Lay Jr D., McMunn K., Cheng H.W., Pajor E., Marchant-Forde R. (2009). Postnatal piglet husbandry practices and well-being: The effects of alternative techniques delivered separately. J. Anim. Sci..

[21] Saller A.M., Werner J., Reiser J., Senf S., Deffner P., Abendschön N., Weiß C., Fischer J., Schörwerth A., Miller R. (2020). Local anesthesia in piglets undergoing castration—A comparative study to investigate the analgesic effects of four local anesthetics on the basis of acute physiological responses and limb movements. PLoS ONE.

[22] Sheil M.L., Chambers M., Sharpe B. (2020). Topical wound anaesthesia: Efficacy to mitigate piglet castration pain. Aust. Vet. J..

[23] Weary D.M., Braithwaite L.A., Fraser D. (1998). Vocal response to pain in piglets. Appl. Anim. Behav. Sci..

[24] Taylor A.A., Weary D.M., Lessard M., Braithwaite L. (2001). Behavioural responses of piglets to castration: The effect of piglet age. Appl. Anim. Behav. Sci..

[25] White R.G., DeShazer J.A., Tressler C.J., Borcher G.M., Davey S., Waninge A., Parkhurst A.M., Milanuk M.J., Clemens E.T. (1995). Vocalization and physiological response of pigs during castration with or without a local anesthetic. J. Anim. Sci..

[26] Marx G., Horn T., Thielebein J., Knubel B., Von Borell E. (2003). Analysis of pain-related vocalization in young pigs. J. Sound Vib..

[27] Puppe B., Schön P.C., Tuchscherer A., Manteuffel G. (2005). Castration-induced vocalisation in domestic piglets, Sus scrofa: Complex and specific alterations of the vocal quality. Appl. Anim. Behav. Sci..

[28] Bilsborrow K., Seddon Y.M., Brown J., Waldner C., Stookey J.M. (2016). An investigation of a novel behavioural test to assess pain in piglets following castration. Can. J. Anim. Sci..

[29] Zankl A. (2007). Untersuchungen zur Wirksamkeit und Gewebeverträglichkeit von Lokalanästhetika bei der Kastration männlicher Saugferkel. Ph.D. Thesis.

[30] Enz A., Schupbach-Regula G., Bettschart R., Fuschini E., Burgi E., Sidler X. (2013). Experiences with pain control during piglet castration in Switzerland Part 1: Inhalation anesthesia. Schweiz. Arch. Tierheilkd..

[31] Patil I. {ggstatsplot}: ‘ggplot2’ Based Plots with Statistical Details. https://CRAN.R-project.org/package=ggstatsplot.

[32] Bates D., Mächler M., Bolker B., Walker S. (2015). Fitting linear mixed-effects models using lme4. J. Stat. Softw..

[33] Lenth R., Buerker P., Herve M., Love J., Riebl H., Singmann H. Estimated Marginal Means, aka Least-Squares Means. https://github.com/rvlenth/emmeans.

[34] Koo T.K., Li M.Y. (2016). A guideline of selecting and reporting intraclass correlation coefficients for reliability research. J. Chiropr. Med..

[35] Taylor A.A., Weary D.M. (2000). Vocal responses of piglets to castration: Identifying procedural sources of pain. Appl. Anim. Behav. Sci..

[36] Kluivers-Poodt M., Hopster H., Spoolder H.A.M. (2007). Castration under Anaesthesia and/or Analgesia in Commercial Pig Production.

[37] Ranheim B., Haga H.A., Ingebrigtsen K. (2005). Distribution of radioactive lidocaine injected into the testes in piglets. J. Vet. Pharmacol. Ther..

[38] Becker D.E., Reed K.L. (2006). Essentials of local anesthetic pharmacology. Anesth. Prog..

[39] Zink W., Graf B.M., Tonner P.H., Hein L. (2011). Lokalanästhetika. Pharmakotherapie in der Anästhesie und Intensivmedizin.

[40] Strichartz G.R., Sanchez V., Arthur G.R., Chafetz R., Martiny D. (1990). Fundamental properties of local anesthetics. II. Measured octanol: Buffer partition coefficients and pKa values of clinically used drugs. Anesth. Analg..

[41] Brau M.E., Vogel W., Hempelmann G. (1998). Fundamental properties of local anesthetics: Half-maximal blocking concentrations for tonic block of Na+ and K+ channels in peripheral nerve. Anesth. Analg..

[42] Davis K., Seddon Y., Creutzinger K., Bouvier M., Brown J. (2017). An investigation into the use of sucrose to reduce castration pain in piglets. Can. J. Anim. Sci..

[43] Zankl A., Ritzmann M., Zöls S., Heinritzi K. (2007). The efficacy of local anaesthetics administered prior to castration of male suckling piglets. DTW Dtsch. Tierarztl. Wochenschr..

[44] Telles F.G., Luna S.P.L., Teixeira G., Berto D.A. (2016). Long-term weight gain and economic impact in pigs castrated under local anaesthesia. Vet. Anim. Sci..

