# Supplementary material for: Local Anesthesia in Piglets Undergoing Castration—A Comparative Study to Investigate the Analgesic Effects of Four Local Anesthetics Based on Defensive Behavior and Side Effects
Source: Animals (Basel). 2020 Sep 26;10(10):1752. doi: 10.3390/ani10101752 (PMC7601579; doi:10.3390/ani10101752)
Supplement: Supplementary file 1 [file animals-10-01752-s001.pdf]

animals

MDPI

Table 1. Score used to evaluate the defensive movements according to Leidig et al. [1] during injection, skin incision and severing of the spermatic cord.

parameter	score	description
intensity	0	no movements
	1	moving of one limb
	2	moving of two limbs
	3	moving of three limbs
	4	moving of all four limbs
duration	0	no movements
	1	one single movement
	2	repeated movements (2 – 4)
	3	continuous movements (> 4)

Table 2. Score used to evaluate the castration wounds according to Zankl [2].

parameter	score	description
wound healing	0	without specific findings (dry, fully closed wound)
	1	minor findings (swelling, redness)
	2	open wound
wound secretion	0	no secretion
	1	serous / bloody secretion
	2	purulent secretion
texture and size of the spermatic cord	0	hardly palpable
	1	up to 1 cm, soft to rough and elastic
	2	stronger than 1 cm, soft to rough and elastic
	3	stronger than 1 cm rough or fluctuating

Table 3. Score used to evaluate the postoperative bleeding according to Enz, *et al.* [3].

parameter	score	description
postoperative bleeding	0	no bleeding
	1	slight bleeding, dissemination < 2 cm
	2	severe bleeding, perineal area extensive bloody, dissemination > 2 cm
	3	severe bleeding, perineal area and hind limbs bloodstained

References

1. Leidig, M.S.; Hertrampf, B.; Failing, K.; Schumann, A.; Reiner, G. Pain and discomfort in male piglets during surgical castration with and without local anaesthesia as determined by vocalisation and defence behaviour. *Appl. Anim. Behav. Sci.* **2009**, *116*, 174-178, doi:10.1016/j.applanim.2008.10.004.
2. Zankl, A. Untersuchungen zur Wirksamkeit und Gewebeverträglichkeit von Lokalanästhetika bei der Kastration männlicher Saugferkel. Diss. med. vet., Ludwig-Maximilians-University, Munich, 2007.
3. Enz, A.; Schupbach-Regula, G.; Bettschart, R.; Fuschini, E.; Burgi, E.; Sidler, X. Experiences with pain control during piglet castration in Switzerland Part 1: Inhalation anesthesia. *Schweiz. Arch. Tierheilkd.* **2013**, *155*, 651-659, doi:10.1024/0036-7281/a000530.

© 2020 by the authors. Submitted for possible open access publication under the terms and conditions of the Creative Commons Attribution (CC BY) license (http://creativecommons.org/licenses/by/4.0/).
